# Georgia Commission on Tuberculosis

**Published:** 1904-11

**Authors:** Chas. Hicks

**Affiliations:** Chairman, Dublin, Ga.


### GEORGIA COMMISSION ON TUBERCULOSIS.

BY CHAS. HICKS, M.D., CHAIRMAN,

DUBLIN, GA.

GENTLEMEN: At this the organization meeting of the State Medical Commission on Tuberculosis, in assuming the duties which the action of the Legislature involves, I deem it proper to incorporate in the opening remarks, as a preface, the resolution passed authorizing the Governor to raise this commission:

*Resolved by the Senate, the House concurring,* That the Governor of the State of Georgia be, and he is hereby, authorized to raise a Medical Commission to consist of one physician from each Congressional District, and ten from the State at large, who shall make a thorough investigation touching the propositions hereinafter set forth and numbered, and embody the same in a report to be submitted to the next session of the General Assembly of Georgia. (1) The number of cases of tuberculosis in the State of Georgia. (2) The annual number of deaths from tuberculosis in the State of Georgia. (3) The number of cases contracted within the State, and the number imported. (4) The measures that are being taken in the various localities for preventing the spread of the disease. (5) Whether or not the physicians of the State are in favor of taking measures for its prevention.

*Be it further resolved,* That said Commission shall serve without compensation and without entailing any expense whatever upon the State of Georgia in connection with said investigation.

This act of the Legislature, taken in connection with the fact that this same Legislature did not see fit to grant the recommendation of the State Board of Health for an appropriation sufficient to procure proper clerical aid to secure to the Department of Health the required and absolutely necessary statistics, places this Commission in an anomalous attitude. Justice demands that the report from this Commission to the next General Assembly should deal frankly with this feature of the State's draft on the medical profession. The State Board of Health is not in a position to furnish the statistics required of us, nor is it probable that the Board will be in position to do so before a report from this Commission will have been due at the capitol.

Correct statistics can be secured only through legally authorized health organizations, with power to act, and which have been provided with means to procure necessary data. Not only is it true that the State Board of Health has not been properly provided for financially, but there are many counties and municipalities throughout the State that have not organized local boards of health. The law creating the State Department of Health contemplated, and the ordinances of the State Board of Health provide for, the establishment of local boards of health in every county and municipality in the State, which are to be integral parts of the public health system. If these irregularities arise from lack of official concern, complaint should be lodged with the department at fault. If the law does not give specific authority to enforce organization, this Commission, in its report to the Legislature, should emphasize the necessity for additional legislation, to authorize the Executive Department or the Health Department to organize health boards in every county and municipality not having them, and to require the local authorities to properly provide for and equip them. For, without sufficient clerical force and means, the State Board of

Health can no more correctly conduct the business of the Department of Health than could the tax officials collect the dues to the State without properly prepared digests on which to report.

While we have thus far been recounting what the State has or has not done, we can not, nor should we, hold altogether blameless the medical profession for our State's tardiness in securing the blessings to be derived from public health laws. More than half a century ago, not a great while after Georgia merged into the sisterhood of States, the pioneers and patriarchs of medicine in our State organized the Medical Association of Georgia, our sanctum sanctorum, the ark of our covenant, where has ever reigned Science—truth unveiled—the inspiring Diana of all true craftsmen. The cardinal principle in the Association's constitution is: "To secure a high standard of professional qualifications and ethics, and to promote professional brotherhood." It is, therefore, not strange that this organization has maintained for Georgia peers among medical men throughout all the States.

There was another object in the same covenant: "To organize the medical profession of the State in the most efficient manner possible." This latter constitutional obligation has not been fulfilled, hence our inability or inefficiency to comply with the State's request for needed information.

Thus we see demonstrated not only the misgivings of those who have in charge statecraft, but the medical profession's unreadiness. We see the necessity of the most thorough organization possible of the profession to properly perform its duties in the march of progress and to the welfare of the commonwealth—not politically, for a review of Goethe's "Farbenlehre" would be an easy task by the side of giving an epitome, even of what the results would be, of a scientific body organizing politically for politics' sake. The immediate organization of the medical profession should be perfected, so that duties may be efficiently performed and obligations discharged which no other class of citizens can do for the State. Every member of the medical profession within the confines of the State should be brought to account for his worth to the public weal. Out of about four thousand "regulars" practicing in this State, there are not exceeding one-fifth of this number who are members of the

Medical Association of Georgia loyally attending its scientific meetings. This state of affairs encourages charlatanry of high and low degree. Bring the profession together in solid phalanx and purge the State of charlatanry—the *ignis fatuus*, alluring the unfortunate and weak into the quagmire of venal quackery, from which it arises! Such results would be wholesome in the work we have met today to discuss and plan to execute. A memorial should go from this body to the officers of the Medical Association asking that the resolution offered at its last meeting, relative to county organizations, be held of force and perfected without delay.

In the matter of organization, we are not without precept and example in our State. Who is it that does not recall twenty years ago, when the Agricultural Society of Georgia could scarce find encouragement or place to hold its fair. Within the gates of this city today the farmers of Georgia are placing on exhibition the finest display in the history of their organization. At an international show in St. Louis, at this hour, the Georgia State Agricultural Department is challenging the world with the products of our State's forests and fields and mineral resources. Through organization the agriculturists have let their needs be known and made properly their demands. If one needs proof of results, read the annual budget of the Legislature and see the provision for the State Agricultural Department. Nor is this all. Under the auspices of the State University, organization and teaching are kept up through the Farmer's Institute in every county in the State, educating along the lines of scientific farming. The Bulletin Georgia Department of Agriculture, Serial No. 41, season 1903-1904, is a marvel of proficiency, betokening the deserved success of the department. The Commissioner of Agriculture, in commending the contents of his report, aptly quotes Dean Swift: "He that maketh two ears of corn or two blades of grass grow upon a spot of ground, where only one grew before, deserves better of mankind and does more essential service to his country than the whole race of politicians put together."

Since twenty-five years ago, when the silk-growers, wine-makers and brewers of France engaged M. Pasteur to investigate the difficulties in producing these commodities, the agriculturist has become a new Richmond in the field of science. Touched by the wand of science, difficulties have fallen and disappeared before the eyes of the agriculturist, and today he is a new factor in the affairs of state, adding to his frugality by uniting science, through a well-appointed State department, with his energies, broadening the possibilities of his commonwealth. In turn, the commonwealth has properly placed at the farmer's disposal the means to encourage his enterprise and advance agriculture. The law does not permit fertilizers below standard grade to be sold in Georgia; every pound must be the choicest combinations for crops—pure food for vegetation. Appropriations are made for experimental stations, experts are sent all over the State to examine into and to exterminate and eradicate blight, scale and diseases among vineyard, orchard, field and herd; and to meet the threatening invasion of the boll weevil, the preparation is none the less pretentious than were the demonstrations for war in our State during our common country's late unpleasantness with Spain.

Whether the encouragement given the Agricultural Department be the promptings of thrift or of economy, the State should *a fortiori* equip and empower the Department of Health for the betterment of her citizenry. Having passed the experimental stage, the medical profession can assure the State of the curability and the preventability of infectious and contagious diseases. Especially is this true, though strange the statement may seem to some, of the particular subject in hand—Tuberculosis. Consumption, which Oliver Wendell Holmes has appropriately called "The White Plague," is not hereditary, but strictly infectious; is not only preventable and curable, but one of the most easily cured of the chronic diseases. There is no specific treatment for consumption. Drugs advertised and not advertised will not cure it. But plenty of good food, pure water, comfortable clothing, proper environment, with plenty of fresh air and abundance of sunshine, together with the necessary precautionary measures to prevent the spread of the bacilli only, meet the indications for the cure of tuberculosis. Not longer dreaming theories, but reasoning by induction, has gradually pushed back the veil of ignorance until synthetical philosophy has yielded up the truth of conditions, and by the application of the knowable laws, consumption has lost its position at the head of the list, in fatalities, of the diseases which have been scourges of the human race.

Germany, the pioneer in making a State fight against tuberculosis, has persistently waged war on the bacillus tuberculosis, and through the health department, philanthropy, and the assistance of life-insurance agencies, can point to two score or more of mammoth sanatoria, curing annually thousands of consumptives.

In this country, in recent years, the State of Massachusetts erected at Rutland the first sanatorium for the exclusive treatment of tuberculosis. More recently New York, New Jersey, Iowa, Maine and Illinois have taken steps in this direction, and it is believed that within ten years every State in the Union will have its own consumptive sanatoriums. Within twenty years, in the city of New York, the death-rate from consumption has been reduced forty-one per cent. At this rate, the next generation of men will see eradicated from that State "the great white plague." The States with rigorous winters have to erect expensive buildings, with glass houses attached, for sun baths. Our State, in a temperate climate, can, by the use of tents and starting at the sands of the seashore, march with the sun up the Atlantic slope, across "the red hills of Georgia" to the crest of the Blue Ridge and back again, furnish at reasonable cost sanatorium facilities and a solarium the year round in the open air.

To realize results in our work, the public must be educated to the necessities, for which we should invoke the aid of the teachers, the clergy, philanthropy, and a generous public press.

"We must care for the consumptive in the right place, in the right way, and at the right time, until he is cured; instead of, as now, in the wrong place, in the wrong way, at the wrong time, until he is dead."—PRYOR.

"If every individual in his respective sphere, and the local, State and Federal governments, would do its full duty in the combat of this fearful scourge of mankind, I am convinced that before many decades tuberculosis would be eradicated from our midst, and the United States would have the honor of being the first among the nations of the earth to have accomplished this great and glorious work."—KNOPF.

